# [Retracted] Upstream transcription factor 1 prompts malignancies of cervical cancer primarily by transcriptionally activating p65 expression

**DOI:** 10.3892/etm.2024.12631

**Published:** 2024-07-01

**Authors:** Wen Wang, Shujuan Yao, Hongjing Jiang, Jing Dong, Xiujuan Cui, Xiangyu Tian, Yanyan Guo, Shiqian Zhang

Exp Ther Med 16:4415–4422, 2018; DOI: 10.3892/etm.2018.6758

Following the publication of this paper, it was drawn to the Editors’ attention by a concerned reader that certain of the cell migration and invasion assay data shown in Fig. 2 and the western blotting data shown in Fig. 3B on p. 4419 were strikingly similar to data appearing in different form in a pair of other articles written by different authors at different research institutes that had already been published elsewhere prior to the submission of this paper to *Experimental and Therapeutic Medicine*. In view of the fact that the abovementioned data had already apparently been published previously, the Editor of *Experimental and Therapeutic Medicine* has decided that this paper should be retracted from the Journal. The authors were asked for an explanation to account for these concerns, but the Editorial Office did not receive a reply. The Editor apologizes to the readership for any inconvenience caused.

